# Building a knowledge base for colorectal cancer patient care using formal concept analysis

**DOI:** 10.1186/s12911-021-01728-y

**Published:** 2022-11-23

**Authors:** Jing Xiang, Hanbing Xu, Suresh Pokharel, Jiqing Li, Fuzhong Xue, Ping Zhang

**Affiliations:** 1 School of Public Health and Management, Binzhou Medical University, Yantai, China; 2 Department of Biostatistics, School of Public Health, Shandong University, Jinan, China; 3 School of Information Technology and Electrical Engineering, The University of Queensland, Brisbane, Australia; 4 Menzies Health Institute Queensland, Griffith University, Gold Coast, Australia

**Keywords:** Colorectal cancer, Knowledge base, Patient similarity, Formal concept analysis, Concept retrieval

## Abstract

**Background:**

Colorectal cancer (CRC) is a heterogeneous disease with different responses to targeted therapies due to various factors, and the treatment effect differs significantly between individuals. Personalize medical treatment (PMT) is a method that takes individual patient characteristics into consideration, making it the most effective way to deal with this issue. Patient similarity and clustering analysis is an important aspect of PMT. This paper describes how to build a knowledge base using formal concept analysis (FCA), which clusters patients based on their similarity and preserves the relations between clusters in hierarchical structural form.

**Methods:**

Prognostic factors (attributes) of 2442 CRC patients, including patient age, cancer cell differentiation, lymphatic invasion and metastasis stages were used to build a formal context in FCA. A concept was defined as a set of patients with their shared attributes. The formal context was formed based on the similarity scores between each concept identified from the dataset, which can be used as a knowledge base.

**Results:**

A hierarchical knowledge base was constructed along with the clinical records of the diagnosed CRC patients. For each new patient, a similarity score to each existing concept in the knowledge base can be retrieved with different similarity calculations. The ranked similarity scores that are associated with the concepts can offer references for treatment plans.

**Conclusions:**

Patients that share the same concept indicates the potential similar effect from same clinical procedures or treatments. In conjunction with a clinician’s ability to undergo flexible analyses and apply appropriate judgement, the knowledge base allows faster and more effective decisions to be made for patient treatment and care.

## Background

Colorectal cancer (CRC) is a malignant and immunogenic tumor disease in the digestive tract. Globally, it is the second most common cancer in women and the third most commonly occurring cancer in men, causing more than 500,000 deaths every year [1, 2]. Chemotherapy, radiotherapy and surgery are the common treatments for colorectal cancer. Individual heterogeneity of sensitivity and toxicity within and between tumors leads to significantly different treatment effects for each individual, despite the same treatment being applied on the same tumor site. Personalize medical treatment (PMT) is one of the most effective solutions for this problem. PMT is the tailoring of treatments based on individual patient characteristics (biological features and environmental factors). In the case of CRC, the aim of PMT is to effectively avoid potential adverse effects or delay the adverse effects until a better treatment alternative is found [3].

Cancer staging is the process of finding out how much cancer is in a person’s body and where it’s located. Along with the type of cancer a person has, the stage of the cancer is one of the most important factors for doctors to determine a patient’s prognosis and treatment. A tumor staging system, named TNM [4], is mostly used for prognosis of colorectal cancer. In the TNM system, the overall stage is determined after the cancer is assigned a letter or number categories, to describe the original tumor (T), the spread to the lymph node (N), and whether the cancer has spread/metastasized (M). The "T" plus a letter or number (0 to 4) is used to describe how deeply the primary tumor has grown into the bowel lining. The original tumor stage may also be divided into smaller groups that help describe the tumor in even more detail. Lymph nodes are small, bean-shaped organs located throughout the body that help the body to fight infections as part of the immune system. Lymph nodes near the colon and rectum are called regional lymph nodes. All others are distant lymph nodes that are found in other parts of the body. The “N” followed by a number or letter represents whether the tumor cells have spread to the regional lymph nodes, and by how much. For example, N0 represents no spread to regional lymph nodes, N1 means that there are tumor cells found in 1 to 3 regional lymph nodes, and N2 means 4 or more regional lymph nodes. Doctors assign the stages (eg. Stage I, IIA, IIB etc.) of the cancer by combining the T, N, and M classifications to help with patient treatment planning [5]. Pathological staging of CRC has important clinical significance for choosing proper patient treatments and corresponding follow-up plans.

However, even patients with the same stage and pathological type can have different responses to the same radial or chemotherapy regimen, which indicates individual differences among the patients. Similarity analysis of colorectal cancer patients based on their characteristics may help to categorize patients to different groups and recommend the relevant treatment scheme towards personalized treatment [6]. One of the fundamental challenges for PMT is to group patients in subsets (small cluster of patients) based on their similarity and as well as capturing the relationships between them. The key to creating meaningful patient clusters is to capture the right patient characteristics (features) and to apply the suitable algorithms. Two important tasks involved in the process are (i) similarity computation: it measures the similarity between the CRC patients based on their characteristics such as cancer stage, vital signs, symptoms and other clinical signs, and (ii) capturing relationships: the relationships can be captured at the patient level as well as between the subsets created based on the common characteristics shared by the group of patitents. The use of patient clusters enables us to build a knowledge base to assist with making clinical applications.

In the past, many methods have been proposed for patient similarity computation, utilizing cancer staging levels and other prognostic factors. Huang et al. [7] proposed a patient similarity measurement to build predictive models for diabetes status. The similarity calculation method was based on Euclidean distance and Jaccard distance. Pai et al. [8] presented a novel supervised patient classification framework based on patient similarity networks-netDx, the similarity metric used was Euclidean distance and Pearson correlation. In the same scope, Pokharel et al. [9] used an ontology-based method for calculating patient similarity for intensive care unit (ICU) patients. Pokharel et al. [10, 11] later continued to propose temporal tree with sequential pattern mining to capture inherent relationships between the clinical events due to their co-occurrence. However, all the above-mentioned patient similarity-based methods only focused on computing the similarity between the patients and did not consider preserving the relationships into the patient level and at the subsets level.

To address the problems listed above, we proposed the formal concept analysis (FCA) based similarity computation approach. FCA expresses knowledge bases in a hierarchical structure, which also reflects the relations between concepts or clustered groups [12]. Each concept in the hierarchy represents corresponding objects that share a set of properties, and each sub-concept in the hierarchy represents a subset of the objects (as well as a superset of the properties) in the concept above it. This research aims to build a knowledge base for colorectal cancer patients using FCA. The concept lattice included in the knowledge base can be used as references for individual patient treatment planning.

## Methods

### Data sets

The data used for this study were collected for our earlier study [13] from the hospital information system (HIS) of Shandong provincial hospital, China from November 2010 and July 2016, and it was made available for this study and future validation of algorithms. The main inclusion criteria for selecting the patient cohort were: (i) diagnosed as CRC based on histology or cytology, (ii) at least 18 years old, (iii) had not received neoadjuvant therapy (iv), received radical surgery, (v) available for follow-up data, (vi) preoperative biochemical test data can be obtained. Furthermore, patients were not included in the study if they met any of the following exclusion criteria: (i) urgent and untreatable due to widespread metastasis, (ii) have been diagnosed with other additional cancers and (iii) currently receiving preoperative chemotherapy or immunotherapy. After selecting patients based on the inclusion and exclusion criteria, there were 2442 eligible patients diagnosed with CRC.

Besides cancer stages, patient age, cancer cell degree of differentiation, histological type, number of sample lymph nodes, carcinoembryonic antigen (CEA), cancer antigen 19-9 (CA19-9), lymph node ratio (LNR) and lymph vascular invasion have been reported as prognostic factors of cancer patients [13–17]. Each of these values were recorded for each patient in the collected data set and were used to build a knowledge base in this study. The number of layers of original tumor that has grown into the wall of the colon or rectum were also recorded in the dataset, including T1, T2, T3 and T4. Spread of regional lymph nodes were recorded as N0, N1 and N2. Seven cancer stage groups were formed based on the TNM system, stage I, IIA, IIB, IIC, IIIA, IIIB and IIIC, each representing different levels of cancer spread into nearby tissues or lymph nodes [5].

### Formal concept analysis

The term formal concept analysis was first introduced by Wille in 1981 [18]. It provides a conceptual framework for structuring, analyzing and visualizing data, to make them more understandable [11]. The method defines a concept as a unit comprising a set of objects and a set of their shared attributes. It identifies all concepts and their dependencies from the tabular input data which is defined as a formal context. Two sets of output data are produced from the analysis performed on the formal context. The first output provides a hierarchical relationship of all the established concepts in the form of a line diagram called a concept lattice (Hasse diagram). In the concept lattice, each concept is represented as a node, referred to as the concept node. The second set of outputs is a list of all the interdependencies found among attributes in the formal context [19]. In FCA, every concept in the concept lattice (a node in the line diagram) consists of two parts: connotation (attribute set) and extension (object set). The concept lattice explains the relationship between connotation and extension, and it is the unity of the connotation and the extension.

1. ***Formal context***

A formal context in FCA can be represented as a triple *K* = *(G,M,I*), where *G* and *M* are two sets of elements and represent objects and attributes, respectively. *I* is the binary relationship between *G* and *M*. In order to express the relationship *I* between object *g* and attribute *m*, we can write it as *gIm* or $\left(g,m\right)\in I$ and interpret it as "object *g* has attribute *m*". In formal context *K* = *(G,M,I),* given a set of object subsets $A\subseteq G$ and a set of attribute subse*ts* $B\subseteq M$, then a set of dual operators *A'* and *B'* are defined as follows:

1 $$\begin{aligned} A^{\prime} & = \left\{ {m \in M{|}\forall g \in A, gIm} \right\} \\ B^{\prime} & = \left\{ {g \in M{|}\forall m \in B, gIm} \right\} \\ \end{aligned}$$

where *A'* is a collection of all the attributes that are shared by all the objects in *A*, and *B'* is a collection of all the objects, of which each object has all the attributes in *B*. If *A* and *B* satisfy *A'* = *B* and *B'* = *A*, then (*A*, *B*) is called a concept of formal context, where *A* is the extension of the concept and *B* is the connotation of the concept [18].

**Fig. 1:**
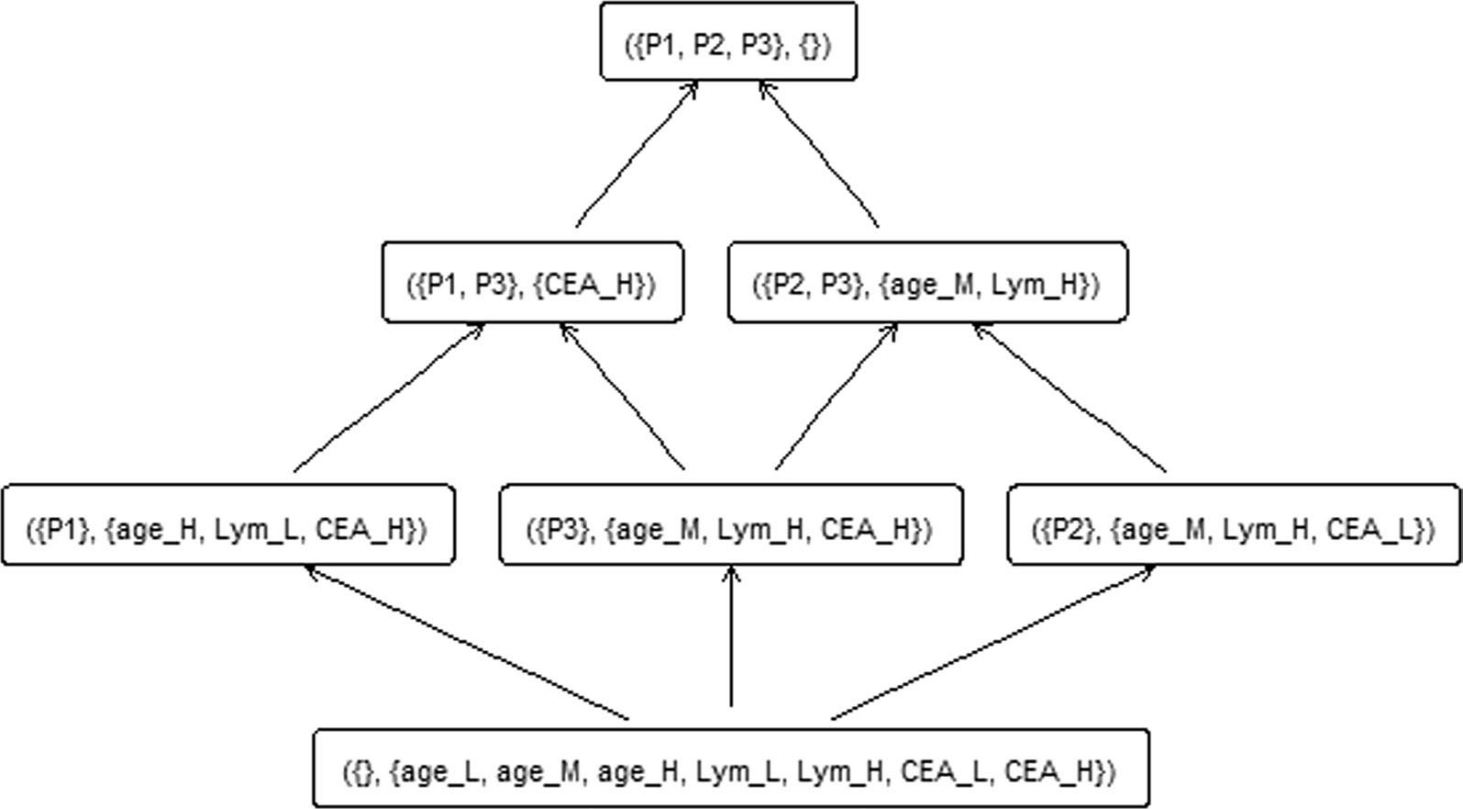
Context lattice created based on the example dataset that includes 3 patients and 6 attributes (Table 1)

In this study with the colorectal cancer data, each patient is an object, and the corresponding features (characteristics) of patients are attributes. We establish the formal context according to the relations between the sets of objects and attributes, thus forming a hierarchical structure among concepts, which is a concept lattice. To further explain how the formal context is established, we use only 3 of our patients (3 objects) with 6 features (6 attributes) as an example dataset to create a visualisable context. The example dataset includes 3 patients with each patient having 6 attributes age_L, age_M, age_H, Lym_L, Lym_H, CEA_L, CEA_H (Table 1). The characteristics of each patient are represented by the attributes with a binary value. For example, patient1’s age is over 70 years old (age_H), low in lymph nodes number (Lym_L) and high in carcinoembryonic antigen level (CEA_H). With this example dataset, the whole set of objects G includes 3 patients noted as P1, P2 and P3. M is the whole set of attributes Age_L, Age_M, Age_H, Lym_L, Lym_H, CEA_L and CEA_H. The subset of the attributes {Age_M, Lym_H} is shared by objects P2 and P3, and {P2, P3} is the collection of all the objects that share the 2 attributes. Then ({p2, p3}, {Age_M, Lym_H}) is a concept. Also, {P1, P3} is a collection of all the objects that shared the attribute CEA_H, this makes ({P1, P3}, {CEA_H}) a concept. With this example dataset, 5 concepts can be created. The whole concept lattice (context) created with this dataset can be visualised in Fig. 1. Inside the context, each node represents a concept.

2) ***Concept similarity in FCA***

**Table 1:** Example Dataset with 3 Patients and 6 Attributes

Object	Attributes*						
	Age_L	Age_M	Age_H	Lym_L	Lym_H	CEA_L	CEA_H
P1	0	0	1	1	0	0	1
P2	0	1	0	0	1	1	0
P3	0	1	0	0	1	0	1

*Age_L: age < 60, Age_M: age 60 to 70, Age_H: age > 70; Lym_L, lymph nodes < 10; LymH, lymph nodes ≥ 10

*CEA_L* carcinoembryonic antigen level low, *CEA_H* carcinoembryonic antigen level high

Concept similarity can be measured by the distances between the concepts in the hierarchy of concept lattice. At present, there are many methods to calculate concept similarity, such as concept similarity based on concept instances, concept similarity based on attributes and concept similarity based on concept relations.

Concept similarity indicates the degree at which two concepts share the same objects and attributes. The closer two entity concepts are (sharing more objects and attributes), the higher the concept similarity they will have. The calculation of similarity between concept nodes can be measured by distance. A greater distance between two concept nodes indicates a lesser number of the same objects and attributes being shared between the two concept nodes, that is, the concept similarity is lower. Concept similarity between two concept nodes (*A*_*1*_, *B*_*1*_ and *A*_*2*_, *B*_*2*_) can be calculated as follows:

2 $$\begin{gathered} {\text{Sim(}}(A_{1} ,B_{1} ),(A_{2} ,B_{2} )) \hfill \\ = \left( {\frac{{\left| {A_{1} \cap A_{2} } \right|}}{m}} \right)*\alpha + \left( {\frac{{\left| {B_{1} \cap B_{2} } \right|}}{n}} \right)*\beta \hfill \\ \end{gathered}$$

where *m* = *max ((A*_*1*_*|, |A*_*2*_*|), n* = *max (|B*_*1*_*|, |B*_*2*_*|).* When the objects and attributes of concept nodes are equally considered, α = β = 0.5.

For retrieving similar concepts from the context that share attributes with a new object (in this study a patient), we calculated the similarity between the patient and the concepts based on attributes, which is the case of formula (2) with α = 0, β = 1.

## Results

### Description of patients’ characteristics

The characteristics of the patients included in this study is shown in Table 2. From the total of 2442 patients, 1108 (45.4%) patients were younger than 60 years old, 824 (33.7%) patients were 60–70 years old, and 216 (21.6%) patients were older than 70 years old. Most of the patients (N = 1878 or 76.9%), have moderate degree of cancer cell differentiation. Majority of the patients (N = 2115 or 86.6%) were recorded as adenocarcinoma. Over 90 percent of the patients (N = 2218) had more than 10 lymph nodes. The preoperative carcinoembryonic antigen level of 1487 (60.9%) patients was lower than 5 ng/mL, and preoperative cancer antigen level of 2100 patients (86.0%) was lower than 37 U/mL. More than 95 percent of the patients (N = 2333 vs N = 109) did not have lymphovascular invasion, and 1799 (72.9%) patients had lymph nodes ratios lower than 0.13. T1, T2, T3 and T4 tumor stages accounted for 128 (5.2%), 388 (15.9%), 592 (24.2%) and 1334 (54.6%) of patients, respectively. Lymph node stages N0, N1 and N2 accounted for 1489 (61.0%), 533 (21.8%) and 420 (17.2%) patients, respectively. The cancer stage groups in this dataset included I, IIA, IIB, IIC, IIIA, IIIB and IIIC, with patient numbers in each stage of 428 (17.5%), 396 (16.2%), 648 (26.5%), 17 (0.7%), 66 (2.7%), 530 (21.7%) and 357 (14.6%), respectively.

**Table 2:** Baseline statistical description of patients with CRC

Characteristic	N (%)
**Age (years)**	
< 60	1108 (45.4)
60–70	824 (33.7)
≥ 70	510 (20.9)
**Cell differentiation**	
moderate	425 (17.4)
poor	1878 (76.9)
well	139 (5.7)
**Histological type**	
Mucinous adenocarcinoma	327 (13.4)
Adenocarcinoma	2115 (86.6)
**Lymph nodes**	
< 10	224 (9.2)
≥ 10	2218 (90.8)
**CEA (μg/ml)**	
< 5	1487 (60.9)
≥ 5	955 (39.1)
CA19-9 (U/ml)	
< 37	2100 (86.0)
≥ 37	342 (14.0)
**Lymphovascular invasion**	
Yes	109 (4.5)
No	2333 (95.5)
LNR	
< 0.13	1779 (72.9)
≥ 0.13	663 (27.1)
**T stage**	
T1	128(5.2)
T2	388(15.9)
T3	592(24.2)
T4	1334(54.6)
**N stage**	
N0	1489(61.0)
N1	533(21.8)
N2	420(17.2)
**Cancer stage group**	
I	428 (17.5)
IIA	396 (16.2)
IIB	648 (26.5)
IIC	17.0 (0.7)
IIIA	66.0 (2.7)
IIIB	530 (21.7)
IIIC	357 (14.6)

### Formal context of colorectal cancer patients

The formal context of the 2442 patients was constructed with the fcaR package in R [20]. Attributes (features) of patients were coded as binary values before being used as the input of the package. Part of the input file is shown in Table 3. A total of 31,741 concepts were obtained from the data set, including the top one which does not contain any attributes and the bottom one that has an empty set of objects. Table 4 shows some of the concept values in the formal context. Each row in the table represents one concept in the context, with its corresponding attributes and the number of objects included in the concept. The concepts at the top of the concept lattice hierarchy include more individual patients who share the small sets of attributes, while the concepts at the lower levels contain less patients who share more specific attributes. The last four rows of the table also show the patients included in the concepts as examples, when the number of objects is not more than 2.

**Table 3:** Colorectal Cancer Patients and Attributes

Object	Attributes*															
	Age _L	Age _M	Age _H	Degre_L	Degre_M	degre_H	MAd	Ad	T1	T2	T3	T4	N0	N1	N2	Lym_L
Patient1	0	0	1	1	0	0	1	0	0	0	0	1	0	0	1	1
Patient2	0	1	0	0	1	0	0	1	0	0	0	1	1	0	0	0
**…**	**…**	**…**	**…**	**…**	**…**	**…**	**…**	**…**	**…**	**…**	**…**	**…**	**…**	**…**	**…**	**…**
Patient1000	0	0	1	0	1	0	0	1	0	0	1	0	0	0	1	0
Patient10001	0	0	1	1	0	0	0	1	1	0	0	0	1	0	0	0
**…**	**…**	**…**	**…**	**…**	**…**	**…**	**…**	**…**	**…**	**…**	**…**	**…**	**…**	**…**	**…**	**…**
Patient2441	1	0	0	1	0	0	1	0	0	0	0	1	0	1	0	0
Patient2442	0	1	0	0	1	0	0	1	1	0	0	0	1	0	0	0

Object	Lym _H	CEA _L	CEA _H	Ca199_L	Ca199_H	Ly _N	Ly _Y	LNR _L	LNR _H	I	IIA	IIB	IIC	IIIA	IIIB	IIIC
Patient1	0	0	1	1	0	1	0	0	1	0	0	0	0	0	0	1
Patient2	1	1	0	0	1	1	0	1	0	0	0	1	0	0	0	0
**…**	**…**	**…**	**…**	**…**	**…**	**…**	**…**	**…**	**…**	**…**	**…**	**…**	**…**	**…**	**…**	**…**
Patient1000	1	0	1	1	0	1	0	0	1	0	0	0	0	0	1	0
Patient10001	1	1	0	1	0	1	0	1	0	1	0	0	0	0	0	0
**…**	**…**	**…**	**…**	**…**	**…**	**…**	**…**	**…**	**…**	**…**	**…**	**…**	**…**	**…**	**…**	**…**
Patient2441	1	1	0	1	0	1	0	1	0	0	0	0	0	0	1	0
Patient2442	1	1	0	1	0	1	0	1	0	1	0	0	0	0	0	0

*Age_L: age < 60, Age_M: age 60 to 70, Age_H: age > 70; Degre_L: degree of cell differentiation (differentiation level) poor, Degre_M: degree of cell differentiation moderate, Degre_H: degree of cell differentiation well; MAd: mucinous adenocarcinoma, adenocarcinoma; Lym_L: lymph nodes < 10, LymH: lymph nodes ≥ 10; CEA_L: carcinoembryonic antigen level low, CEA_H:carcinoembryonic antigen level high; Ca199_L: cancer antigen 19-9 low, Ca199_H: cancer antigen 19-9 high; Ly_N: no lymph vascular invasion, Ly_Y: lymph vascular invasion occurred: 0: No, 1: Yes

**Table 4:** Concepts in the context of colorectal cancer patients

Concept	Attribute set	Number Of Objects
C2	IIIB	530
C3	LNR_H	663
C4	LNR_H, IIIB	278
C5	LNR_L	1779
C6	Lv_Y	109
C7	Lv_Y, IIIB	31
C8	Lv_Y, LNR_H	66
C9	Lv_Y, LNR_L	43
C10	Lv_N	2333
……	……	……
C31736	Age_L, Degre_L, MAd, T3, N0, Lym_H, CEA_H, Ca199_H, Lv_N, LNR_L, IIA	1 (1823)**
C31737	Age_L, Degre_L, MAd, T3, N0, Lym_H, CEA_H, Ca199_L, Lv_N, LNR_L, IIA	2 (549, 831)**
C31738	Age_L, Degre_L, MAd, T3, N0, Lym_H, CEA_L, Ca199_L, Lv_N, LNR_L, IIA	2 (411,569)**
C31739	Age_L, Degre_L, MAd, T2, N0, Lym_L, CEA_L, Ca199_L, Lv_N, LNR_L, I	1 (980)**
C31740	Age_L, Degre_L, MAd, T1, N0, Lym_H, CEA_L, Ca199_L, Lv_N, LNR_L, I	1 (772)**

*Age_L: age < 60, Age_M: age 60 to 70, Age_H: age > 70; Degre_L: degree of cell differentiation (differentiation level) poor, Degre_M: degree of cell differentiation moderate, Degre_H, degree of cell differentiation well; MAd, mucinous adenocarcinoma, adenocarcinoma; Lym_L, lymph nodes < 10, LymH, lymph nodes ≥ 10; CEA_L, carcinoembryonic antigen level low, CEA_H, carcinoembryonic antigen level high; Ca199_L, cancer antigen 19-9 low, Ca199_H, cancer antigen 19-9 high; Ly_N, no lymph vascular invasion, Ly_Y, lymph vascular invasion occurred

**Within the brackets are the IDs of the patients, for example, 549 represents patient549

The lattice (the context) with all the patients and attributes constructed can be used as a knowledge base. We hypothesize that patients who share the same concept indicates the potential of similar effect with same clinical procedures or treatments. For example, the set of objects in concept 9 (C9) included 43 patients. These patients share the properties, with evidence of lymphovascular invasion and high lymph nodes ratios, and therefore may share similarities in clinical procedures or treatment plans.

### Concept retrieval for new colorectal cancer patients

**Fig. 2:**
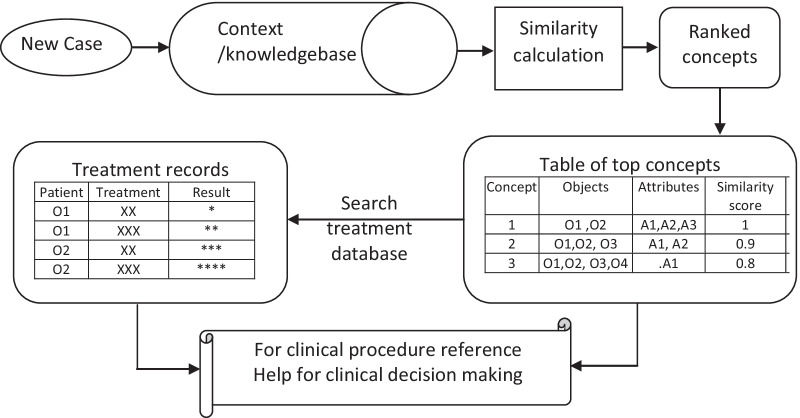
Information retrieval from the knowledge base for a new patient (O1, O2 are examples of objects, and A1, A2 and A3 are examples of attributes)

In this study, each patient along with its all attributes can be considered as a concept. To retrieve the concepts that have high similarity with the new patient concept, similarity between the new case and the concepts within the context was calculated based on patient attributes described earlier. When there exists a concept in the knowledge base (context) that share the whole set of attribute values with the new patient, a concept that matches to the new patient 100% can be retrieved. The rest of the concepts can be ranked by the similarity levels between each concept and the new patient case (Fig. 2). Table 5 shows the top similar concepts retrieved for a new patient that shares the same attributes as patient 980. The similarity scores between the new case and the concepts within the context were calculated with formula (2).

**Table 5:** Similarity scores (top 10) retrieved from the context, for a new patient who shares all the attributes with patient 980

Concept	Objects	Attributes	Similarity score
C31739	980	Age_L, Degre_L, MAd, T2, N0, Lym_L, CEA_L, ca199_L, Lv_N, LNR_L, I	1.000
C31719	2425	Age_L, Degre_L, MAd, T4, N0, Lym_L, CEA_L, ca199_L, Lv_N, LNR_L, IIB	0.818
C31740	772	Age_L, Degre_L, MAd, T1, N0, Lym_H, CEA_L, ca199_L, Lv_N, LNR_L, I	0.818
C31718	436, 474, 725, 759, 1074, 1146, 1761, 2248	Age_L, Degre_L, MAd, T4, N0, Lym_H, CEA_L, ca199_L, Lv_N, LNR_L, IIB	0.727
C31738	411, 569	Age_L, Degre_L, MAd, T3, N0, Lym_H, CEA_L, ca199_L, Lv_N, LNR_L, IIA	0.727
C31728	411, 569, 2279	Age_L, Degre_L, MAd, T3, Lym_H, CEA_L, ca199_L, Lv_N, LNR_L	0.636
C31732	2279	Age_L, Degre_L, MAd, T3, N1, Lym_H, CEA_L, ca199_L, Lv_N, LNR_L, IIIB	0.636
C31734	411, 549, 569, 831	Age_L, Degre_L, MAd, T3, N0, Lym_H, ca199_L, Lv_N, LNR_L, IIA	0.636
C31737	549, 831	Age_L, Degre_L, MAd, T3, N0, Lym_H, CEA_H, ca199_L, Lv_N, LNR_L, IIA	0.636
C31723	411, 549, 569, 831, 2279	Age_L, Degre_L, MAd, T3, Lym_H, ca199_L, Lv_N, LNR_L	0.545

*Age_L: age < 60; Degre_L: degree of cell differentiation (differentiation level) poor; Mad, mucinous adenocarcinoma, adenocarcinoma; Lym_L, lymph nodes < 10, LymH, lymph nodes ≥ 10; CEA_L, carcinoembryonic antigen level low, CEA_H, carcinoembryonic antigen level high; Ca199_L, cancer antigen 19-9 low, Ca199_H, cancer antigen 19-9 high; Lv_N, no lymph vascular invasion, Lv_Y, lymph vascular invasion occurred

As we hypothesized that shared and distinguished attributes by the concepts may offer some insight to clinicians. With the treatment record of each patient included in the concepts with high similarity, a more suitable treatment plan may be suggested for a new patient.

## Discussion

In this study, FCA is introduced for patient similarity analysis which not only presents a hierarchy, but also reflects the relationship between concepts. The concept lattice built from this study can be used as a reference knowledge base for choosing alternative treatment plans for the patients who did not receive an effective treatment. For example, patient A and patient B share one concept. However, patient A had effective treatment, and patient B had another treatment procedure but not as effective as patient A had. Doctors can then review the treatment plan for patient A and adjust the treatment for patient B accordingly.

A similarity measure, between a new patient as a new case and the existing patients along with their attributes as concepts within the knowledge base, can be used to help find the patients who share the most attributes with the new patient. In this study, the similarity between patients or the similarity between a new case and a concept included in the formal context was calculated based on patients’ attributes as described in formula 2. The similarity can be calculated by different methods, for example Euclidean distance or the method proposed by Tadrat et al. [21] and suggested by Finnie and Sun [22]. We hypothesize that patients with high similarity in attributes or patients who are within the concepts close to each other should share certain clinical values. A group of concepts that have high similarity to each other can offer more expanded insight to assist with making clinical decisions. Different levels of concept clustering may be applied for different clinical practices. In this study we calculated similarity scores between a new case and the concepts within the built context for case retrieval, using one similarity calculation algorithm. We have not created concept clusters within the context, and this will be our future study to test different similarity algorithms when we have enough following up patient treatment records for further validation.

## Conclusions

In this study, the concept lattice theory was applied to patient similarity analysis. A context of CRC patients was built with extra patient attributes in addition to cancer stages. It can be used as a knowledge base for clinicians to provide alternative clinical advice to new or existing patients. For each patient, a similarity score to each existing concept in the lattice can be retrieved. Along with the clinician’s ability to analyze with flexibility and pass judgment, the knowledge base offers significant support to doctors in providing appropriate advice and making decisions. Further clustering based on the concepts presented in the lattice can be applied to various clinical applications, corresponding to different patient categorizations. The concept lattice theory adds value in health services, elevating personalized cancer care or treatment.

## Data Availability

The dataset used for this study is stored on the research storage server at Griffith University (https://research-storage.griffith.edu.au/owncloud). It is available for authorised users or on request to the corresponding author.

## Abbreviations

CA19-9: Cancer antigen 19-9
CRC: Colorectal cancer
FCA: Formal concept analysis
LNR: Lymph node ratio
PMT: Personalize medical treatment
TNM: A tumor staging system (tumor, lymph nodes and metastasis
